# The epidemiology of low vision and blindness associated with trichiasis in southern Sudan

**DOI:** 10.1186/1471-2415-7-12

**Published:** 2007-08-28

**Authors:** Jeremiah Ngondi, Mark Reacher, Fiona Matthews, Francis Ole-Sempele, Alice Onsarigo, Ibrahim Matende, Samson Baba, Carol Brayne, Paul Emerson

**Affiliations:** 1 Department of Public Health and Primary care, Institute of Public Health, University of Cambridge, Cambridge, UK; 2 MRC Biostatistics Unit, Institute of Public Health, Cambridge, UK; 3 Christian Mission Aid, Nairobi, Kenya, Africa; 4 Family Health International, Nairobi, Kenya, Africa; 5 Lighthouse For Christ Eye Centre, Mombasa, Kenya, Africa; 6 Ministry of Health, Government of Southern Sudan, Juba, Sudan, Africa; 7 The Carter Center, Atlanta, GA, USA

## Abstract

**Background:**

We investigated vision status associated with trachomatous trichiasis (TT) and explored age-sex patterns of low vision and blindness associated with trichiasis in Mankien district of southern Sudan where trachoma prevention and trichiasis surgery were absent.

**Methods:**

A population based survey was undertaken and eligible persons underwent eye examination. Visual acuity (VA) was tested using Snellen E chart and persons with TT identified. Vision status was defined using the WHO categories of visual impairment based on presenting VA: normal vision (VA ≥ 6/18 in better eye); low vision (VA < 6/18 but ≥ 3/60 in better eye); and blindness (VA < 3/60 in better eye). An ordinal logistic regression model was fitted and age/sex specific distribution of vision status predicted.

**Results:**

Overall 341/3,567 persons examined had any TT. Analysis was based on 319 persons, 22 persons were excluded: 20 had both TT and cataract; and 2 had missing VA data. Of the 319 persons: 158(49.5%) had trichiasis-related corneal opacity (CO); bilateral TT and bilateral CO were found in 251(78.7%) and 110 (34.5%), respectively; 146 (45.8%) had low vision or blindness; the ratio of low vision to blindness was 3.2:1; and no sex differences were observed. In our model the predicted distribution of vision status was: normal vision, 53.9% (95% CI 50.9–56.9); low vision, 35.3% (95% CI 33.3–37.2); and blindness, 10.9% (95% CI 9.7–12.0).

**Conclusion:**

We have reported severe trichiasis and high prevalence of vision loss among persons with trichiasis. Our survey showed that almost 1 in 20 of the entire population suffered low vision or blindness associated with trachoma. The need for trichiasis surgery, trachoma prevention services, and rehabilitation of the blind is acute.

## Background

The World Health Organization (WHO) estimates that 1.3 million people world wide are blind due to trachoma [1] and 7.6 million have trichiasis, the potentially blinding stage of the disease, in 55 countries [2]. In southern Sudan, the magnitude and prevalence of active trachoma and trachomatous trichiasis (TT) are among the highest in the world. In areas surveyed so far, mean prevalence of active trachoma in children aged 1–9 years was 64% while trichiasis prevalence in persons aged 15 years and above was 9.2%, thus making blinding trachoma a severe public health problem [3]. Trichiasis is generally accepted to commence in the second and third decades of life with up to 10% of adults in endemic communities being affected [4-6]. However, early onset of trichiasis has been observed in southern Sudan with a mean trichiasis prevalence of 1.4% in children aged under 15 years and individuals as young as four years affected [3,7].

Recurrent infection with ocular *Chlamydia trachomatis *results in chronic inflammation, scarring, trichiasis, and corneal opacification [8-10]. Trachomatous blindness is due to irreversible corneal damage arsing from physical corneal abrasion, corneal infections and defective tear film [4,5]. Blindness mainly results from corneal opacity (CO) that obscures the visual axis, and is graded on examination of the eye by the presence of an opacity which is dense enough to obscure the pupil margin. Although vision loss has also been observed in individuals with peripheral corneal opacity [11] trichiasis alone, in the absence of overt corneal opacity, can result in substantial visual impairment and disability [12]. Previous studies estimating the global burden of trachoma have indicated paucity of data on trachomatous low vision and blindness and suggest the need for population based studies to estimate low vision and blindness arising from trachoma [13-15]. The objective of this study was to investigate vision status in persons with trichiasis and to explore the age and sex patterns of low vision and blindness associated with trichiasis.

## Methods

### Study setting and participants

This study was conducted in Mankien payam (district) of southern Sudan. The sample size and sampling plan has been described previously [7,16]. In brief, a population based survey was conducted to estimate the prevalence of blindness, low vision, active trachoma and trichiasis. A two-stage cluster random sampling design was used to select the sample: villages were selected in stage one and households selected in stage two. A total of 3,567 persons who were present in the selected households underwent eye examination. The analysis presented here comprises persons of all ages and sexes presenting with any TT.

### Ocular examination

Clinical examination of the eyes was undertaken by qualified integrated eye care workers (IECWs) who had been trained in visual acuity testing and trachoma grading by an experienced ophthalmic nurse. The examiners' reliability was validated with the ophthalmic nurse as the gold-standard. Only examiners who had an inter-observer agreement of at least 80% were eligible to participate in the survey.

#### Visual acuity testing and basic eye examination

Prior to the survey, the minimum age for visual acuity (VA) testing was predetermined to be 5 years. VA testing was conducted outdoors in adequate sunlight using the Snellen E chart at 6 meters. In persons with VA < 6/60, VA was evaluated with the Snellen chart at 3 meters. Further VA assessment was done in persons with VA < 3/60 by counting fingers, hand movement and light perception as appropriate. All participants then underwent basic eye examination. Using a torch and a ×2.5 magnifying binocular loupe, each eye was examined first for in-turned lashes (TT), and the cornea was then inspected for corneal opacities (CO), and the lens examined for cataract. TT was defined as the presence of at least one eyelash touching the eyeball or evidence of epilation of eyelashes; CO was defined as easily visible opacity that obscured the papillary margin; and cataract was defined as an opacity of the lens that appeared greyish or white when examined with oblique light, under a shaded area [17].

Data were recorded on a customized form and the cause of visual impairment determined for all subjects with a presenting VA of < 6/18 for each eye separately. The principal disorder responsible low vision or blindness was determined for the participant by taking into account the main cause for each individual eye. Vision loss was attributed to trachoma in persons presenting with trichiasis and corneal opacity. In the instance where different causes of vision loss had been identified for each eye separately in a given individual, the principal disorder was chosen to be the one that was most readily curable or, if not curable, most easily preventable (i.e. cataract, trachoma, non-trachomatous CO, and other causes in that order).

#### Definition of vision status

The WHO categories of visual impairment were used to define individual vision outcomes (vision status) [18]. *Blindness *was defined as a presenting visual acuity of less than 3/60 in the better eye. *Low vision *was defined as presenting visual acuity of less than 6/18 but equal to or greater than 3/60 in the better eye. *Normal vision *was defined to represent persons who had normal or near-normal vision in the better eye (VA ≥ 6/18). An ordinal score of vision status comprising of three categories was then assigned to all eligible participants based on increasing severity of visual impairment: where '1' was normal vision; '2' low vision; and '3' blindness.

### Statistical analysis

Data were double entered by different entry clerks and compared for consistency using EpiInfo version 3.3.2 (CDC, Atlanta, Georgia). Statistical analysis was conducted using Stata 8.2 (Stata Corporation, College Station, Texas). Contingency table analysis was used to examine demographic characteristics. Differences in age/sex distribution and proportions were tested using χ^2 ^test. Age specific distributions of vision status were calculated for 5-year age intervals. We fitted an ordinal logistic regression model to the observed data to explore the age and sex distribution of the three categories of vision status: normal vision; low vision; and blindness [19]. Persons with vision loss not attributable to trichiasis were excluded from the final model. Children aged 0–4 years were assumed to have normal vision. Predicted probabilities were derived to smooth age-specific curves for the three categories of vision status [20].

### Ethical consideration

The Sudan Peoples Liberation Movement Secretariat of Health (SPLM/Health) and the Institutional Review Board of Emory University approved the protocol and clearance to conduct the surveys was obtained from the local authorities. Verbal consent to participate was sought from the head of the household and from each individual and the parents of children aged 10 years and below in accordance with the declaration of Helsinki. Personal identifiers were removed from the data set before analyses were undertaken. Participants who required surgical intervention or further assessment were referred to attend an eye surgical camp that was organised and conducted after the survey.

## Results

### Characteristics of the study participants

**Figure 1:**
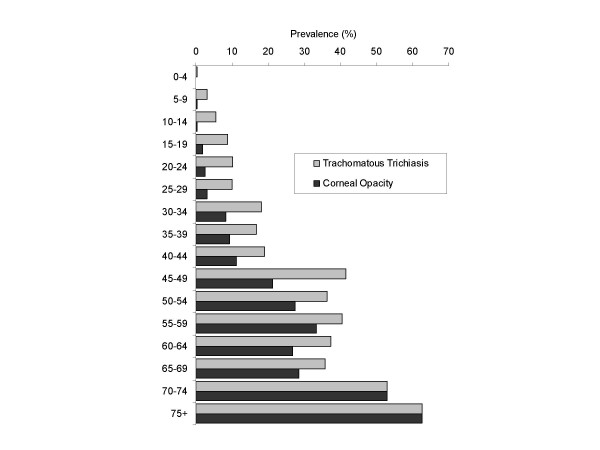
Age specific prevalence of trachomatous trichiasis and corneal opacity (n = 3,567).

**Figure 2:**
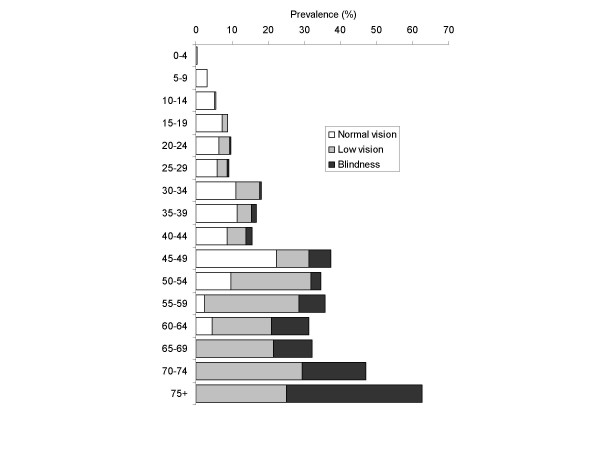
**Age specific prevalence of vision loss attributable to trachoma (n = 3,567)**. Normal vision = presenting visual acuity of ≥ 6/18 in the better eye; Low vision = presenting visual acuity of < 6/18 to ≤ 3/60 in the better eye; Blindness = presenting visual acuity of < 3/60 in the better eye. Three children aged 0–4 years assumed to have normal vision.

**Figure 3:**
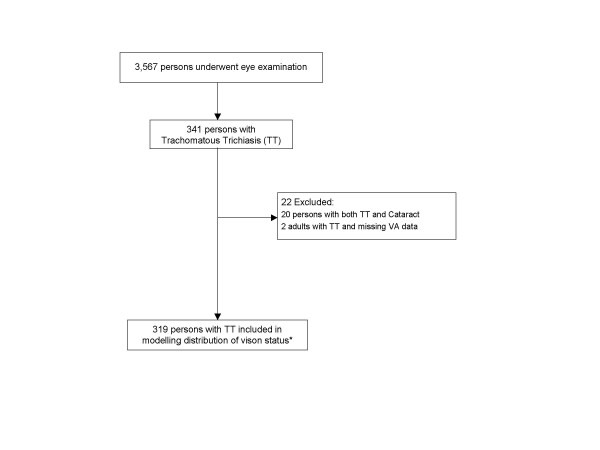
**Sample population**. *Three children aged 0–4 years assumed to have normal vision. VA, visual acuity.

The prevalence of TT, CO, and vision loss attributable to trachoma among the 3,567 people surveyed are shown in Figure 1 and Figure 2. The following analysis relates to people in whom trachomatous trichiasis (TT) was detected. A total of 341 persons (9.6% of 3,567 examined) were found to have any trachomatous trichiasis (TT). Of the 341 persons with any TT, 22 were excluded from the analysis: 20 had both TT and cataract making it impossible to determine which condition was responsible for the visual acuity findings; and 2 had missing visual acuity (VA) data (Figure 3). Six persons with trichiasis and CO in one eye had non-trachomatous CO in the contra-lateral eye and were included in the analysis. Three children with TT aged 0–4 years, who were not eligible for VA examination, were assumed to have normal vision. Prevalence of cataract in the general population was 4.9% (122/2,499) [16], which was not statistically different to prevalence of cataract in the population with TT, which was 5.9% (20/341) (*P = 0.43*).

Among the 319 persons with TT included in the analysis: males and females comprised 117 (36.7%) and 202 (63.3%), respectively; mean age of was 37.2 years (standard deviation = 18.4); and median age was 39 years (inter-quartile range = 21–50). Trichiasis-related corneal opacity (CO) was detected in 159 (49.8%); and bilateral TT and CO were found in 251(78.7%) and 110 (34.5%), respectively. The proportion of persons with TT presenting with CO increased with age.

### Distribution of vision status

#### Observed distribution of vision status

The observed distribution of vision status is shown on Table 1. Overall 54.2% (95% CI = 48.6–59.8) had normal vision, 34.8% (95% CI = 29.6–40.3) had low vision, and 11.0% (95% CI = 7.8–14.9) had blindness. There was no sex difference in the overall distribution of observed visual status (*P = 0.95*). In 173 persons presenting with normal vision (VA > 6/18 in the better eye), 46 (26.6%) had visual impairment (VA < 6/18) in the other eye. The severity of visual impairment increased with age with all persons aged 65 years and above presenting with either low vision or blindness.

**Table 1:** Frequency distribution of observed vision status by age group and sex in persons with trichiasis (n = 319)

Age group (years)	Vision status* (n)											
	
	Males				Females				Total			
			
	Normal vision	Low vision	Blindness	Total	Normal vision	Low vision	Blindness	Total	Normal vision	Low vision	Blindness	Total
0–4**	0	0	0	0	3	0	0	3	3	0	0	3
5–9	8	0	0	8	13	0	0	13	21	0	0	21
10–14	8	1	0	9	9	0	0	9	17	1	0	18
15–19	12	1	0	13	8	3	0	11	20	4	0	24
20–24	10	5	1	16	5	2	0	7	15	7	1	23
25–29	1	2	1	4	12	4	0	16	13	6	1	20
30–34	4	3	0	7	16	9	1	26	20	12	1	33
35–39	6	4	0	10	11	2	2	15	17	6	2	25
40–44	1	0	0	1	9	6	2	17	10	6	2	18
45–49	10	3	0	13	12	6	6	24	22	9	6	37
50–54	4	8	0	12	7	17	3	27	11	25	3	39
55–59	0	8	2	10	1	3	1	5	1	11	3	15
60–64	0	2	5	7	3	9	2	14	3	11	7	21
65–69	0	2	1	3	0	4	2	6	0	6	3	9
70–74	0	1	2	3	0	4	1	5	0	5	3	8
75+	0	1	0	1	0	1	3	4	0	2	3	5

Total: n	64	41	12	117	109	70	23	202	173	111	35	319
Total: %	54.7	35.0	10.3		54.0	34.7	11.4		54.2	34.8	11.0	
95% CI	45.2–63.9	26.5–44.4	5.4–17.2		46.8–61.0	28.1–41.7	7.3–16.6		48.6–59.8	29.6–40.3	7.8–14.9	

*Vision status: Normal vision = presenting visual acuity of ≥ 6/18 in the better eye; Low vision = presenting visual acuity of < 6/18 to ≤ 3/60 in the better eye; Blindness = presenting visual acuity of < 3/60 in the better eye.

**Three children aged 0–4 years assumed to have normal vision.

#### Predicted distribution of vision status

**Figure 4:**
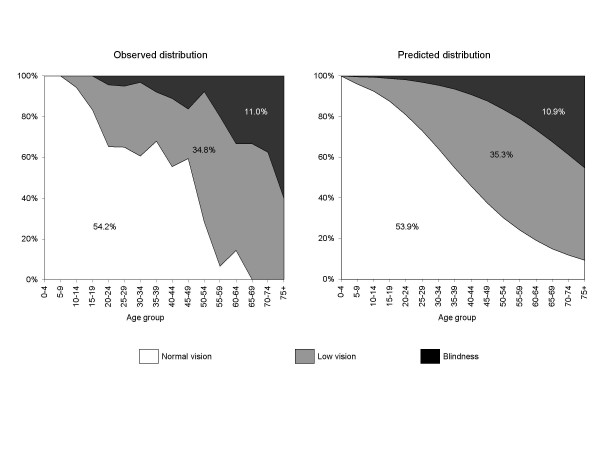
**Observed and predicted distribution of vision status by age group in persons with trichiasis (n = 319)**. Vision status: Normal vision = presenting visual acuity of ≥ 6/18 in the better eye; Low vision = presenting visual acuity of < 6/18 to ≤ 3/60 in the better eye; Blindness = presenting visual acuity of < 3/60 in the better eye. Children aged 0–4 years assumed to have normal vision.

Table 2 and Figure 4 show the predicted distribution of vision status. Overall 53.9 (95% CI = 50.9–56.9) had normal vision, 35.3% (95% CI = 33.3–37.2) had low vision; and 10.9% (95% CI = 9.7–12.0) had blindness. Consistent with the observed data, no sex differences were observed in the overall predicted distribution of vision status (*P = 0.62*) and severity of visual impairment increased with age. The ratio of low vision to blindness was 3.2:1 (95%CI = 2.6:1 to 3.5:1). Sensitivity analysis of distribution of vision status in persons with TT, including those with vision loss due to cataract showed results similar to those presented (data not shown).

**Table 2:** Distribution of predicted proportions of vision status by age group and sex in persons with trichiasis (n = 319)

Age group (years)	Vision status* (%)								
	
	Males			Females			Total		
			
	Normal vision	Low vision	Blindness	Normal vision	Low vision	Blindness	Normal vision	Low vision	Blindness
0–4**	100.0	0	0	100	0	0	100	0	0
5–9	94.6	5.0	0.4	96.3	3.3	0.3	96.0	3.7	0.4
10–14	90.1	9.2	0.7	93.1	6.2	0.7	92.4	6.9	0.7
15–19	83.9	14.8	1.3	88.4	10.4	1.2	87.4	11.4	1.2
20–24	76.1	21.8	2.1	82.2	15.8	2.0	80.9	17.2	2.0
25–29	67.2	29.7	3.2	74.7	22.3	3.0	72.9	24.0	3.1
30–34	57.6	37.7	4.7	66.0	29.4	4.5	64.1	31.4	4.5
35–39	48.2	45.1	6.7	56.9	36.6	6.5	54.8	38.6	6.5
40–44	39.4	51.3	9.3	47.9	43.0	9.1	45.8	45.0	9.1
45–49	31.7	55.7	12.6	39.4	48.2	12.4	37.6	50.1	12.4
50–54	25.2	58.2	16.5	32.0	51.6	16.4	30.3	53.3	16.4
55–59	19.9	58.9	21.2	25.6	53.3	21.1	24.2	54.8	21.1
60–64	15.6	57.9	26.5	20.3	53.1	26.6	19.1	54.4	26.4
65–69	12.2	55.4	32.4	16.0	51.4	32.5	15.1	52.5	32.4
70–74	9.6	51.8	38.6	12.6	48.4	38.9	11.9	49.4	38.7
75+	7.5	47.4	45.0	10.0	44.6	45.5	9.3	45.5	45.2

Total	55.0	35.0	10.0	55.3	33.8	10.9	53.9	35.3	10.9
95% CI	49.6–60.3	31.4–38.7	8.1–11.9	52.4–58.3	31.8–35.7	9.7–12.1	50.9–56.9	33.3–37.2	9.7–12.0

*Vision status: Normal vision = presenting visual acuity of ≥ 6/18 in the better eye; Low vision = presenting visual acuity of < 6/18 to ≤ 3/60 in the better eye; Blindness = presenting visual acuity of < 3/60 in the better eye.

**Children aged 0–4 years assumed to have normal vision.

## Discussion

This study provides a contemporary survey of the relationship between trachomatous trichiasis and visual impairment (low vision and blindness) in southern Sudan. In a trachoma hyper-endemic area of southern Sudan, which was not previously accessible during the civil war, one in 10 persons had trichiasis [7]. Trichiasis was found to be severe in nature in a sample of 319 persons: nearly four fifths had bilateral trichiasis; one in eight were children aged less than 15 years; a third had low vision; and one in 10 had blindness. The study revealed that a half of all persons with trichiasis could be expected to have low vision or blindness by the time they reach their late thirties. This epidemiologic picture is consistent with a severe burden of trichiasis and vision loss at a much younger age than had been appreciated. It is also indicative of lack of trichiasis surgery services in this area, and shows a substantial backlog of trichiasis cases that need to be operated and blind persons who need to be rehabilitated. Determining visual acuity is important in understanding the patterns of visual impairment associated with trichiasis and will assist in planning and priority setting for trichiasis surgery services. The study revealed that over half of the persons with trichiasis had normal vision in the better eye. While resources will undoubtedly be inadequate to provide timely surgery for all, priority should be given to those with some residual vision, since that gives the best chance of saving years-of-useful-sight. Trichiasis in children is most likely predictive of a massive future burden of trachomatous blindness and calls for urgent measures for trachoma control. It is very important to identify and recruit children with trichiasis for surgery and to tailor trichiasis surgery services to cater for the young who may require a general anaesthetic [21].

Our study explored vision loss associated with trichiasis. It is possible that some individuals develop trachomatous CO and vision loss without trichiasis, or CO develops first followed by trichiasis [22]. In addition, some people with few inturned lashes effectively epilate the offending lashes making it difficult to detect trichiasis, thus leading to under estimation of trichiasis prevalence [23]. It may not be possible to distinguish corneal opacity from causes other than trachoma e.g. trauma, measles, Xerophthalmia, herpetic eye disease; but these account for a minor proportion of CO compared to trachoma. In central Tanzania, CO without trichiasis was observed to be more prevalent in women below 35 years [22]. It is probable that in trachoma hyper-endemic settings like Mankien, the majority of corneal opacities or phthisis in persons aged 30 years and above, even in the absence of trichiasis, are due to trachoma. Therefore, estimation of vision loss due to trachoma by assessing related CO in the presence of visible trichiasis on examination is likely to underestimate the burden of blindness attributable to trachoma. We assumed that children aged 0–4 years presenting with trichiasis did not have vision loss. Measurement of visual acuity in this age group is particularly difficult [24]; and most of the available assessment techniques are not practical under field settings. Trichiasis in young children highlights the severity of blinding trachoma in Mankien: further specialised assessment of vision status in these children is suggested.

Consistent with findings from other countries in sub-Saharan Africa [1,25] cataract (41%) was the leading cause of blindness in Mankien [16]. Trachoma accounted for 35% of blindness and two thirds of all forms of vision loss, which by far exceeds that observed in other trachoma endemic settings [16,25]. This probably underscores the way uncontrolled trachoma can ravage inaccessible and underserved communities. In our analysis we excluded persons with both TT and cataract thus demonstrating the pattern of vision loss attributable mainly to trachoma. There was no evidence to suggest that persons with trichiasis were more likely to have cataract compared to the general population. However, it is possible that some of the low vision observed among the population with TT was not caused by the TT since other conditions leading to visual impairment such as refractive error were not controlled for. Our study showed overall distribution of low vision and blindness among trichiasis patients consistent with that observed in The Gambia (38% low vision and 7% blindness)[8] and Ethiopia (39.6% low vision and 10.6% blindness)[26]. However, these two studies were not population based and investigated patients presenting for TT surgery. It is possible that patients presenting for surgery were likely to have more severe trichiasis or vision loss compared to those not presenting, thus resulting in higher proportions of persons with low vision.

We did not find any differences in the distribution of proportions of vision status by sex in patients with trichiasis; however, there were many more women who had trichiasis compared to men. Active trachoma, through re-infection, is an important factor in the pathologic process of progression from TS to TT [9]. Scarring complications have been reported to be higher in women than men in many cross-sectional studies [3,7,10,27,28]. It is believed that greater exposure of women to young children, who are the reservoir for active infection, accounts for the female excess in risk of trachomatous complications. However, exposure to children amongst women may be more important in the development of trachomatous scarring (TS) than progression of TS to TT and CO [29]. The severe nature of trachomatous vision loss in Mankien possibly results from early scarring with subsequent progression to trichiasis and rapid progression of vision loss in persons with trichiasis. This alarming epidemiological picture results from the complex interaction between the host and environmental factors. Nonetheless, the specific host and environmental factors that predispose people in southern Sudan, especially children, to such overwhelming levels of blinding trachoma merit further investigation. The ultimate intervention goal for TT surgery is to reduce the prevalence of TT cases to less than 1/1,000 total population [30]. Nonetheless, until the prevalence of active trachoma is reduced for a sustained period, thus eliminating incident cicatrizing trachoma, trachoma will continue to be an important cause of vision loss in southern Sudan.

At present, assessment of CO is used by programmes as the main proxy outcome indicator for vision loss related to trachoma. However, CO is a serious end-stage complication of trachoma and is not a suitable sign for triggering public health intervention. It is, therefore, appropriate to routinely measure visual acuity in patients with trichiasis to prioritise individuals most likely to benefit from surgery. Visual acuity monitoring for post operative follow-up is essential. Deterioration of vision could be used as an indicator to strongly advocate for surgical intervention in patients refusing surgery. Visual acuity is also a valuable outcome in evaluating the impact of surgical intervention since the primary purpose of trichiasis surgery is to prevent vision loss.

## Conclusion

We have reported severe trichiasis and high prevalence of trachomatous vision loss among persons with trichiasis. A half of all persons with trichiasis could be expected to have low vision or blindness by the time they reach their late thirties. The study revealed that nearly 90% of persons with trichiasis have either normal or low vision in the better eye; and therefore trichiasis surgery in likely to be sight saving for them. There is need to urgently implement trichiasis surgery services. In addition, challenges of providing TT surgery including poor uptake, recurrence and provision of general anaesthetic for small children will need to be addressed. Routine assessment of vision status is recommended to assist in planning for trichiasis surgery services in addition to evaluating the success of surgical interventions in preventing vision loss. Antibiotic treatment, facial cleanliness and environmental change should also be implemented to ensure sustained reduction of active trachoma and elimination of incident cicatrizing trachoma and trachomatous vision loss.

## Competing interests

The author(s) declare that they have no competing interests.

## Authors' contributions

All authors participated in designing the study. JN and FOS collected the data, JN analyzed the data. FM supervised data analysis. JN, MR and FM wrote the paper; FOS, AO, IM, SB, CB and PE contributed to editing the paper. All authors read and approved the final manuscript.

## Pre-publication history

The pre-publication history for this paper can be accessed here:


